# Mother to Offspring Transmission of Chronic Wasting Disease in Reeves’ Muntjac Deer

**DOI:** 10.1371/journal.pone.0071844

**Published:** 2013-08-14

**Authors:** Amy V. Nalls, Erin McNulty, Jenny Powers, Davis M. Seelig, Clare Hoover, Nicholas J. Haley, Jeanette Hayes-Klug, Kelly Anderson, Paula Stewart, Wilfred Goldmann, Edward A. Hoover, Candace K. Mathiason

**Affiliations:** 1 Department of Microbiology, Immunology and Pathology, Colorado State University, Fort Collins, Colorado, United States of America; 2 Biological Resource Management Division, National Park Service, Fort Collins, Colorado, United States of America; 3 Department of Veterinary Clinical Sciences, University of Minnesota, Saint Paul, Minnesota, United States of America; 4 The Roslin Institute and Royal (Dick) School of Veterinary Studies, University of Edinburgh, Easter Bush, Midlothian, Scotland, United Kingdom; University of Maryland School of Medicine, United States of America

## Abstract

The horizontal transmission of prion diseases has been well characterized in bovine spongiform encephalopathy (BSE), chronic wasting disease (CWD) of deer and elk and scrapie of sheep, and has been regarded as the primary mode of transmission. Few studies have monitored the possibility of vertical transmission occurring within an infected mother during pregnancy. To study the potential for and pathway of vertical transmission of CWD in the native cervid species, we used a small cervid model–the polyestrous breeding, indoor maintainable, Reeves’ muntjac deer–and determined that the susceptibility and pathogenesis of CWD in these deer reproduce that in native mule and white-tailed deer. Moreover, we demonstrate here that CWD prions are transmitted from doe to fawn. Maternal CWD infection also appears to result in lower percentage of live birth offspring. In addition, evolving evidence from protein misfolding cyclic amplification (PMCA) assays on fetal tissues suggest that covert prion infection occurs *in utero*. Overall, our findings demonstrate that transmission of prions from mother to offspring can occur, and may be underestimated for all prion diseases.

## Introduction

Transmissible spongiform encephalopathies (TSEs), or prion diseases, are fatal neurodegenerative diseases that affect a variety of species, including humans (Kuru; variant Creutzfeldt-Jakob disease [vCJD]), cattle (bovine spongiform encephalopathy [BSE]), sheep (scrapie), mink (transmissible mink encephalopathy [TME]), domestic and nondomestic cats (feline spongiform encephalopathy [FSE]) and cervids (chronic wasting disease [CWD]).

The transmission of prions, while remaining poorly understood, has obvious public health significance and requires sustained re-appraisal of present concepts for both TSE spread and intervention strategies. It is clear that TSEs are spread by horizontal means of transmission via oral ingestion (Kuru, BSE, vCJD, TME, FSE, CWD) and environmental (scrapie, CWD) contact with infectious prions, as well as by medical means including blood transfusion (vCJD, CWD, scrapie), or iatrogenic exposure (vCJD, sCJD). While less studied, supporting evidence has emerged for an additional path of transmission– that from mother to offspring.

A variant form of the human prion disease CJD, known as variant CJD (vCJD), was attributed to the ingestion of BSE-infected meat products [1], [2]. It is known that vCJD can be transmitted from human to human via blood transfusion from both symptomatic and asymptomatic donors [3]–[6] and that a prolonged asymptomatic incubation period occurs in 129 Met/Val individuals [7]. It is estimated that up to 1 in 4,000 individuals in the UK are asymptomatic carriers of vCJD [8]–[10].

To date, 125 children have been born to women who later developed CJD [11]. This is concerning because PrP^CJD^ has been detected in the fetal and pregnancy related tissues of a woman infected with CJD [12]. Although decades may pass before the onset of clinical effects associated with such transmission due to a long subclinical carrier state, the probability that these individuals harbor infectious prions remains high.

Animal TSEs suggest similar disease patterns. Scrapie prions have been detected in maternal and fetal sheep tissues [13]–[22], and there is a higher incidence of clinical disease during the lambing season [14], [23]–[27]. It is known that BSE incidence is higher in calves born to BSE-infected cattle [28], [29] and that BSE has been transmitted to the offspring of BSE-infected female mice expressing bovid PrP [30]. A cheetah cub, born to a FSE-infected cheetah, was housed in a TSE-free environment and fed a TSE-free diet, yet succumbed to FSE at 7 years of age [31], suggesting maternal exposure.

Importantly, infectious prions are present in the brain, bodily fluids and excreta of animals showing no clinical signs of TSE [32]–[37]. For example, the blood, saliva and urine from deer subclinically infected with CWD contain infectious prions [32], [38], [39]. Race and coworkers [40] found a long-term subclinical carrier state in mice infected with hamster scrapie. Atypical BSE (BASE) can be transmitted from non-clinical cattle to primates [41] and cattle exposed to large oral doses of BSE can remain free of clinical signs but are found to harbor infectious prions upon bioassay into bovid PrP transgenic mice [42]. Taken together, these findings suggest that carriers without clinical signs exist and may transmit infection to their offspring via milk, transplacental trafficking or blood.

Chronic wasting disease, known for its efficient transmission among cervids, is now recognized in 22 states, two Canadian provinces and South Korea (http://www.nwhc.usgs.gov/disease_information/chronic_wasting_disease/index.jsp). Studies proved that CWD can be horizontally transmitted among cervids either by direct contact with infected animals [43], their secreta [32], [33], [38], [44] or indirectly through the environment they inhabit [39], [45]. As observed in other animal TSEs, even cervids in the subclinical phase of disease harbor transmissible infectious prions [32], [39], [46]–[49]. Although there is a growing body of evidence that identifies the sources of infectious prions, less defined are the mechanisms by which prions are shed by infected hosts and taken up by their naïve counterparts. One such mechanism may include that from mother to offspring, either *in utero* (vertical transmission), or shortly after birth in colostrum or milk (maternal transmission).

To address the possibility of CWD mother to offspring transmission we utilized Reeves’ muntjac deer (*Muntiacus reevesi*) [50]. This small Asian deer reaches sexual maturity at 6 months of age, is polyestrous, and can be bred and housed conveniently in indoor controlled laboratory conditions, thus providing a suitable *in vivo* model for cervid vertical/maternal transmission studies [51].

We undertook this study to determine: 1) whether Reeves’ muntjac deer are susceptible to CWD, 2) whether CWD is transmitted from mother to offspring, and 3) whether offspring born to CWD-infected mothers develop clinical TSE disease.

## Materials and Methods

### Muntjac Source

All breeding age male and female muntjac were provided by and transported to Colorado State University by Cervid Solutions Inc., Tellico TN. All animals were handled in strict accordance with guidelines for animal care and use provided by the United States Department of Agriculture (USDA), National Institutes of Health (NIH) and the Association for Assessment and Accreditation of Laboratory Animal Care International (AAALAC), and all animal work was approved by Colorado State University Institutional Animal Care and Use Committee (IACUC approval numbers 02-151A, 08-175A and 11-2615A).

### Genotyping

All muntjac dams, fawns and fetuses were genotyped to determine the PRNP coding sequence in the laboratory of Dr. Wilfred Goldmann, The Roslin Institute, University of Edinburgh, Scotland, U.K. DNA amplification and sequencing reactions were performed as described in Hunter et al. [52], but using deer specific PCR primer pair 213d AGGTCAACTTTGTCCTTGGAGGAG and 139u TAAGCGCCAAGGGTATTAGCAT and sequencing primers 86 d CAGTCATTCATTATGCTGCAGACT or 70 u GCTGCAGGTAGATACTCCCTC.

### Biocontainment Protocols

Protocols to preclude extraneous exposure and cross-contamination between cohorts of animals as previously described [38] incorporated protective shower-in requirements, Tyvek™ clothing, masks, head covers and footwear, while maintaining stringent husbandry procedures. Solid and liquid waste from each suite was either incinerated or collected and denatured by alkaline digestion.

### Muntjac Dam Inoculations and CWD Status Monitoring

Muntjac dams were dosed with 1.0 gram brain orally (PO) or 1.0 gram total brain PO/subcutaneously (SC) (0.5 gr PO/0.5 gr SC) sourced from a CWD positive white-tailed deer used in previous studies [38], [53]. Negative control muntjac dams were dosed in the same manner with 1.0 gram brain sourced from naïve white-tailed deer used in previous studies [38]. Dams were monitored for infection by detection of the protease-resistant prion protein associated with CWD (PrP^CWD^), as detected by immunohistochemistry (IHC) of serial tonsil and RAMALT biopsies collected at monthly intervals.

### Breeding

CWD and mock-infected muntjac dams were mated with intact naïve male muntjac to assess the effects of early and late CWD infection on vertical/maternal transmission. Males typically remained with the females for the duration of the pregnancy until the females were isolated prior to parturition. Ultrasounds were performed on all bred muntjac dams at two-week intervals to estimate gestational stage.

### Muntjac CWD Cervid Transgenic (CerTgPrP) Mouse Bioassay

Two cohorts of transgenic mice expressing the cervid prion protein (CerTgPrP) (54) (n = 9/cohort) were intracranially (IC)-inoculated with either 30 µl 0.1% muntjac CWD pool or naïve muntjac obex. The muntjac CWD pool consists of the obex region harvested from two CWD-inoculated dams (#11 and #15) at terminal disease (24 and 26 months post inoculation respectively). Naïve muntjac obex was pooled from two mock-inoculated muntjac (#62 and #64) serving as negative controls. Mice were monitored daily for clinical disease and terminal brain tissues were analyzed for infection by detection of PrP^CWD^ by IHC and Western blot.

### Study Design

#### Dam cohorts

Once the muntjac dams were determined to be CWD lymphoid biopsy positive, two cohorts of CWD-infected muntjac dams and one cohort of negative control muntjac dams were established (Table 1). Cohort 1: dams bred during early stage CWD infection (< ½ time to dam terminal TSE disease), Cohort 2: dams bred during late stage CWD infection (> ½ time to dam terminal TSE disease) and Cohort 3: dams bred as mock-infected negative muntjac.

**Table 1:** Study design to investigate CWD vertical/maternal transmission.

Offspring cohort	Dam’s CWD status	Dam #	Offspring #	Dam’s clinical disease stage (cohort)	Offspring gestational stage
**Full term viable**	Positive	11*	24^2^	Late (2)	Full term
		45	53^1^	Late (2)	Full term
		46	55^1^	Early (1)	Full term
	Negative	16	20^1^	NCD (3)	Full term
		16	22^2^	NCD (3)	Full term
		19	21^1^	NCD (3)	Full term
		19	26^2^	NCD (3)	Full term
**Full term nonviable**	Positive	11*	23^1^	Early (1)	Full term
		50	54^1^	Early (1)	Full term
		15	25^1^	Late (2)	Full term
		47	51^1^	Late (2)	Full term
		44	52^1^	Late (2)	Full term died 10 dpb
		43	56^1^	Late (2)	Full term died 2 dpb
	Negative	16	27^3^	NCD (3)	Full term died 5 dpb
***In utero*** ** harvest**	Positive	47	57^2^	Late (2)	Late gestation
		45	58^2^	Late (2)	Late gestation
		50	59^2^	Late (2)	Early gestation
	Negative	64	80^1^	NCD (3)	Early gestation

* Dam inoculated orally (PO) only. All other dams inoculated PO and subcutaneously (SC).

Late = > ½ time to dam terminal TSE disease.

Early = < ½ time to dam terminal TSE disease.

Late gestation = >3.5 months gestation.

Early gestation = <3.5 months gestation.

NCD = No clinical disease.

dpb = days post birth.

1 Dam’s first conception.

2 Dam’s second conception.

3 Dam’s third conception.

#### Offspring cohort – full-term viable

Three viable offspring were born to CWD-infected dams (Table 1). Two (#24 and #53) were born to CWD-infected dams that conceived during the late stage of infection, while the third (#55) was born to a CWD-infected dam that conceived during the early stage of infection. Two viable offspring (#20 and #26) were born to mock-infected dams and served as negative viable controls. Offspring were left with their mothers for 55 to 150 days post birth (dpb).

#### Offspring cohort – full-term nonviable

Six full-term nonviable offspring were born to CWD-infected dams (Table 1). Two (#23 and #54) were born to dams that conceived during the early stage of CWD-infection; the other four (#25, #51, #52, #56) were born to CWD-infected dams that conceived during late stage infection. Offspring #52 and #56 were born alive, but died at 10 and 2 dpb, respectively. Three offspring born to mock-infected dams served as negative controls. One nonviable negative control offspring (#27) was born and died at 5 dpb, and the other two negative controls were euthanized at 20 and 26 months post birth (mpb) (#21 and #22).

#### Offspring cohort – harvested in utero

Three *in utero* derived fetuses (#57, #58, #59) were harvested from late stage CWD-infected dams euthanized due to terminal clinical disease (Table 1). Fetal tissues were harvested during first (#59), second (#58) and third (#57) trimester based on breeding dates and fetal measurements. Fetuses were classified as early gestation (<3.5 months gestation [#59]) or late gestation (>3.5 months gestation [#58 and #57]). One late stage mock-infected *in utero* harvested fetus (#80) served as negative control.

### Monitoring and Sample Collection

#### Longitudinal monitoring of viable offspring

Viable muntjac offspring were monitored for CWD infection by IHC detection of PrP^CWD^ in serial RAMALT and tonsilar biopsies. Biopsies were collected at birth and thereafter at weekly/monthly intervals using animal specific blades, forceps and biopsy instruments.

#### Terminal collections

Maternal bodily fluids and excreta (blood, saliva, urine, feces), pregnancy related fluids (amniotic and allantoic) and tissues from each dam and fetus (*in utero* harvest) or nonviable offspring muntjac were harvested, fixed and/or frozen for the detection of the PrP^CWD^. Single-use, animal and tissue-specific blades and forceps were used to harvest each tissue.

### Western Blotting

Tissue homogenates were prepared from the obex region of the medulla oblongata at 10% (w/v) in NP-40 buffer (10 mM Tris-HCl buffer pH 7.5, 0.5% NP-40, 0.5% sodium deoxycholate) in a FastPrep FP120 cell disrupter (Qbiogene) with one 45 second cycle at a speed setting of 6.5, then cooled on ice. Homogenates were mixed with proteinase K (PK) (Invitrogen) at a final concentration of 20 µg/ml and incubated at 37°C for 30 minutes with shaking. Samples were mixed with Reducing Agent (10X)/LDS Sample Buffer (4X) (Invitrogen) at a final concentration of 1X, heated to 95°C for 5 minutes, then run through a NuPAGE 10% Bis-Tris gel at 100 volts for 2.5 hours. Proteins were transferred to a polyvinylidene fluoride (PVDF) membrane at 100 volts for 1 hour in transfer buffer (0.025 M Trizma base, 0.2 M glycine, 20% methanol, pH 8.3). The membrane was loaded into a pre-wetted SNAP i.d. blot holder (Millipore) then placed in the SNAP i.d. system (Millipore). Blocking buffer (Blocker Casein in TBS [Thermo Scientific] with 0.1% Tween 20) was added to the blot holder for 10 minutes followed by vacuum removal. Primary antibody BAR224 (Cayman Chemical) conjugated to horseradish peroxidase (HRP) was diluted in blocking buffer to 0.2 µg/ml, added to the blot holder, incubated for 12 minutes, then vacuumed through the membrane. The membrane was washed three times with 30 ml volumes of wash buffer (50% Blocker Casein in TBS, 50% 1X Tris-buffered saline (TBS), 0.1% Tween 20) with continuous vacuum, then developed with ECL Plus Western Blotting Detection Reagents (GE) and viewed on a Luminescent Image Analyzer LAS-3000 (Fujifilm).

### Immunohistochemistry

Tissues were fixed in 10% neutral buffered formalin or paraformaldehyde/lysine/periodate (PLP) for 5 days or 2 days respectively and stored in 60% ethanol. Tissues were treated for 1 hour with formic acid, rinsed with tap water, embedded in paraffin, and 6 µm sections were placed on positively charged slides. Slides were placed in a 65°C incubator for 1 hour to melt the paraffin, run through a series of xylene and graded ethanol baths to deparaffinize and rehydrate the tissue sections, PK digested (20 mg/ml) at 37°C in 1X TBS for 15 minutes, washed for 10 minutes in TNT buffer (0.1 M Tris-HCl, pH 7.5, 0.15 M NaCl, and 0.05% Tween 20), and subjected to heat-induced epitope retrieval in the 2100-Retriever (Prestige Medical) in sodium citrate buffer (0.01 M sodium citrate, 0.05% Tween 20, pH 6.0). Prions were labeled with primary antibody BAR224 (Cayman Chemical) at 1 µg/ml and Envision+ anti-mouse HRP labeled polymer (Dako). Slides were incubated with AEC Substrate-Chromagen (Dako), counterstained with hematoxylin and bluing reagent (0.1% sodium bicarbonate), and coverslipped with an aqueous mounting medium (Vector Laboratories).

### Serial Protein Misfolding Cyclic Amplification (sPMCA)

Fetal tissues were analyzed by sPMCA using previously published methods [32], [54] incorporating the following modifications. One to ten percent tissue homogenates were prepared in 1% TX-100/0.1 M PBS with 2.5 mm ceramic (Zirconium/Silica) beads in a FastPrep FP120 cell disrupter (Qbiogene) with one 45 second cycle at a speed setting of 6.5, then cooled on ice. The initial round of sPMCA incorporated 20 µl of tissue homogenate sample plus 80 µl normal Tg(cerPrP) [55] brain homogenate (NBH) placed in individual PCR tubes (USA Scientific, St. Louis, MO) containing two 2.38 mm and three 3.15 mm Teflon beads (McMaster-Carr). The PCR tubes were sealed with parafilm, vortexed for 6 seconds and subjected to one round of PMCA. Each round of PMCA is equal to 288 cycles of sonication (Misonix, Farmingdale, NY) (10 seconds separated by 5 minute incubations) at 37°C over 24 hours. Five rounds of sPMCA were completed in duplicate per tissue by transferring 20 µl aliquots of the previous round into 60 µl fresh NBH (1∶3 dilution). The fifth round of sPMCA was analyzed by Western blot. The samples were maintained in a blinded format with identities released post Western blot analysis.

## Results

### PRNP Genotypes

For this study the *PRNP* open reading frame was sequenced in full for all muntjac deer (8 dams and 12 offspring). Reeves’ muntjac *PRNP,* as in other cervids, encodes Gln (codon 95), Gly (codon 96), Met (codon 132) and Ser (codon 225) in positions that are known to modulate susceptibility and incubation period of CWD. No polymorphisms were detected in our 20 study animals, and the sequence differed uniquely in codon 98 between *Muntiacus reevesi* (Ser, Genbank accession no. KC788406) and deer from other genera, e.g. mule deer (Thr, *Odocoileus hemionus*, Genbank AY228473) and white-tailed deer (Thr, *Odocoileus virginanus*, Genbank AY286008). Serine at codon 98 is common in ruminants such as cattle, sheep and goats but can also be found substituted by glycine, alanine or threonine in other species.

### Muntjac CWD CerTgPrP Mouse Bioassay

**Figure 1:**
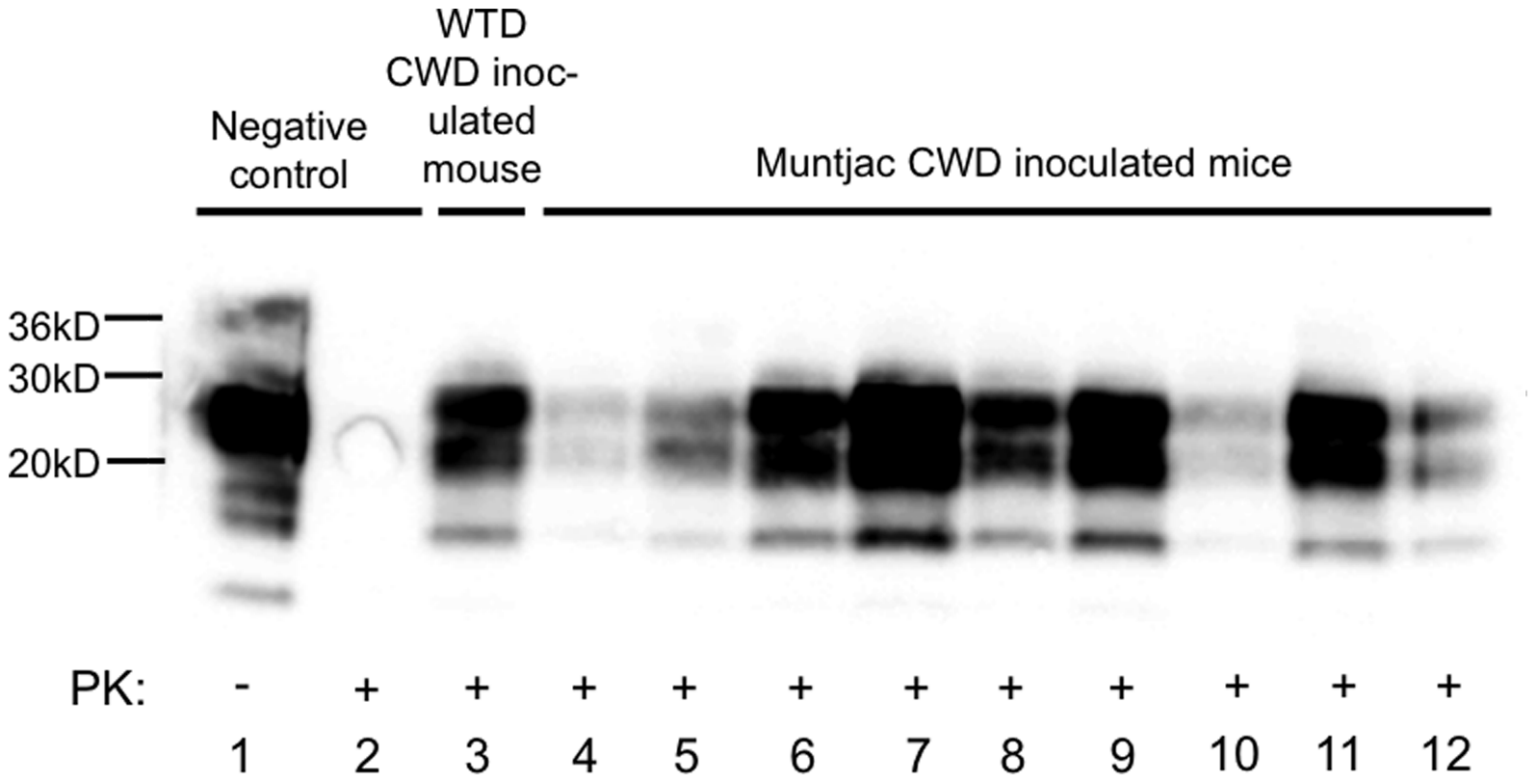
PrP^CWD^ detection in CerTgPrP mice inoculated with muntjac CWD. Western blot detection of PrP^CWD^ in brains of CerTgPrP mice (n = 9) IC-inoculated with obex from a CWD+ white-tailed deer (lane 2) or CWD+ muntjac (lanes 4–12) following PK digestion. Complete PK digestion of PrP^C^ is shown in a mouse inoculated with negative deer obex (lane 2).

Nine of nine muntjac CWD-inoculated mice developed clinical disease including weight loss, hind limb paralysis, stiff tail, and ataxia at 200±29 dpi. CWD detection was confirmed (9/9) and compared to mock-inoculated CerTgPrP mice and white-tailed deer CWD by Western blot analysis (Figure 1).

### Detection of PrP^CWD^ in Muntjac Dams

**Figure 2:**
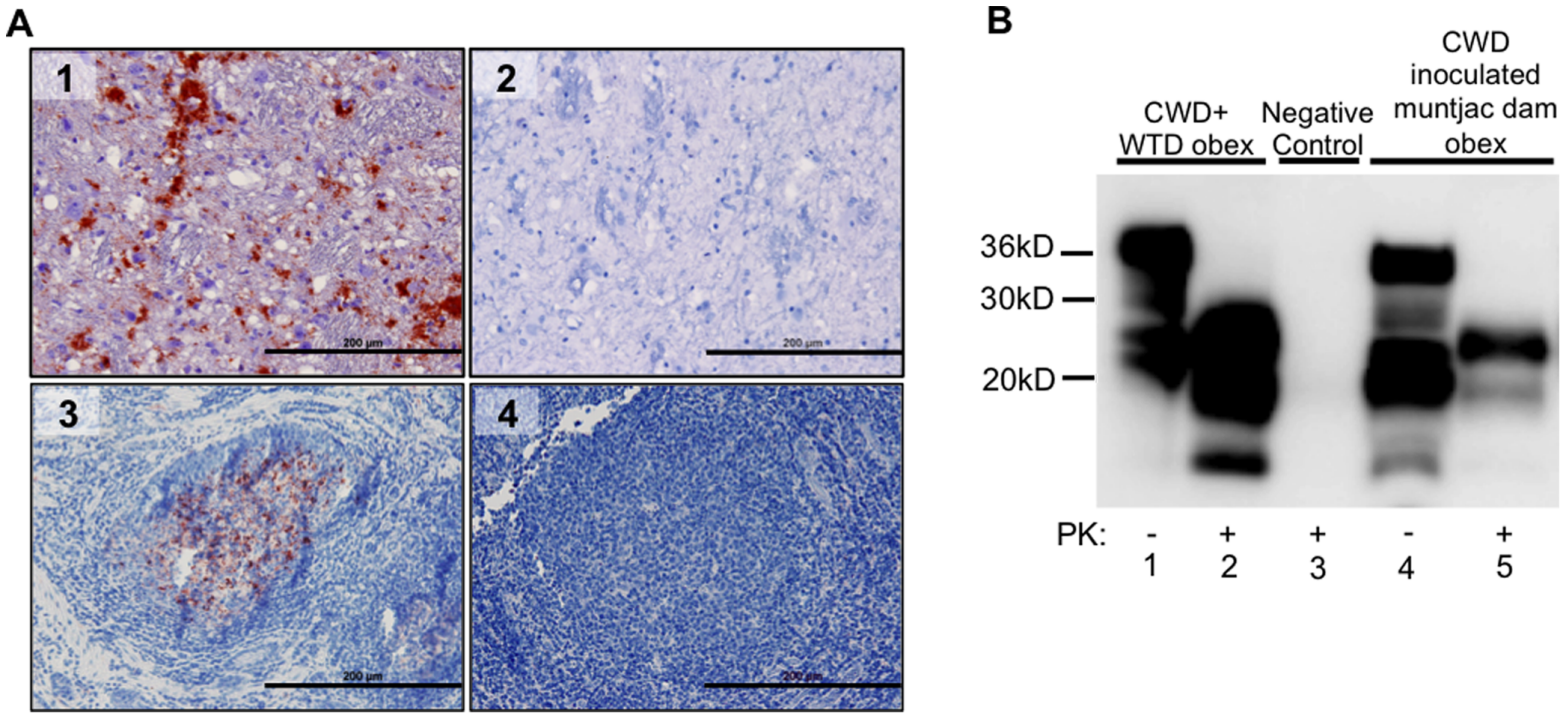
PrP^CWD^ detection in CWD-inoculated dams. (A) IHC PrP^CWD^ is demonstrated (red deposits) in a tonsilar lymphoid biopsy collected at 4 mpi (A3) and in the obex of a terminal TSE diseased dam at 23 mpi (A1). Absence of red deposits is shown in negative control tonsil (A4) and obex (A2). Picture objective is 20X (scale bar = 200 µm). (B) Western blot detection of PrP^CWD^ in obex tissue of a CWD-inoculated dam at 23 mpi (B lane 5) following PK digestion. Complete PK digestion of PrP^C^ is shown in negative control deer obex (B lane 3). PrP^CWD^ detection is demonstrated in CWD positive control deer obex (B lane 2).

Eight of eight CWD-inoculated muntjac dams were immunohistochemistry (IHC) PrP^CWD^ positive in tonsil and/or recto-anal mucosa associated lymphoid tissue (RAMALT) biopsies as early as 4 months pi by IHC (Figure 2A). All dams were confirmed CWD positive by terminal obex IHC and Western blot (Figure 2B).

### Clinical Disease Progression of CWD-positive Muntjac Dams

CWD PO and PO/SC-inoculated dams developed terminal disease including progressive weight and body muscle mass loss, lethargy and ataxia over the course of 18–24 months pi. No appreciable difference was observed between PO or PO/SC inoculates regarding time to biopsy positivity, clinical disease presentation and progression, or the ability to maintain pregnancy. These dams were able to breed and maintain one (4 of 8) or two (4 of 8) full term pregnancies before succumbing to disease. Dams that gave birth to full-term viable and full-term nonviable fawns were able to deliver their offspring naturally without the aid of cesarean section.

### Detection of PrP^CWD^ in Muntjac Offspring (Table 2, Figures 3, 4, 5)

#### Full-term viable offspring

**Figure 3:**
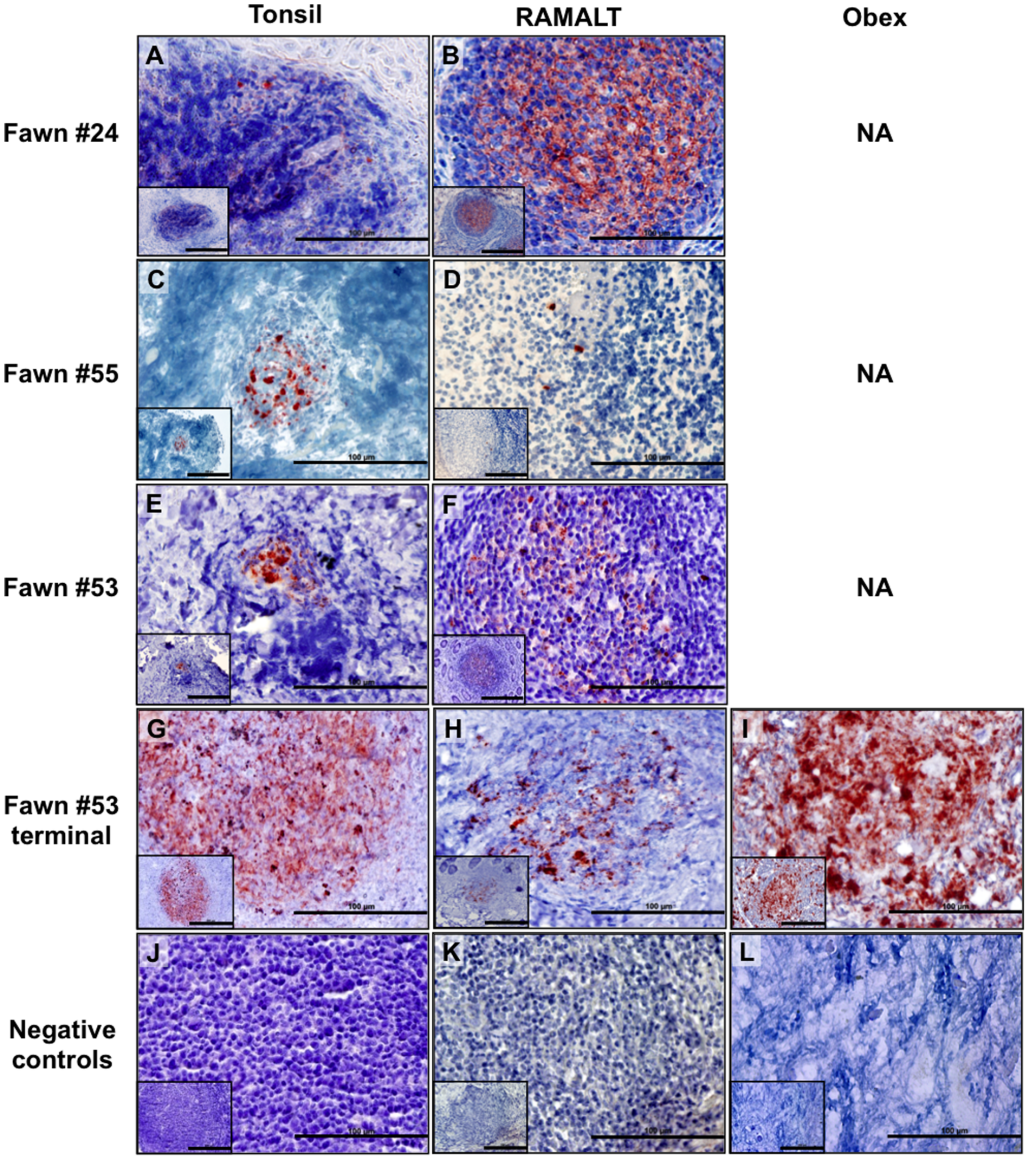
IHC PrP^CWD^ detection in viable muntjac fawns born to CWD+ dams. PrP^CWD^ is demonstrated (red deposits) in tonsilar biopsies collected at 40 days post birth (dpb, A), 465 dpb (C) and 504 dpb (E) and RAMALT biopsies collected at 103 dpb (B), 386 dpb (D) and 504 dpb (F) and confirmed in the terminal tissues of 1 fawn (G, H, I). Absence of PrP^CWD^ is shown in negative control tissues (J, K, L). Picture objective is 40X (scale bar = 100 µm) with insets at 20X (scale bar = 200 µm).

**Figure 4:**
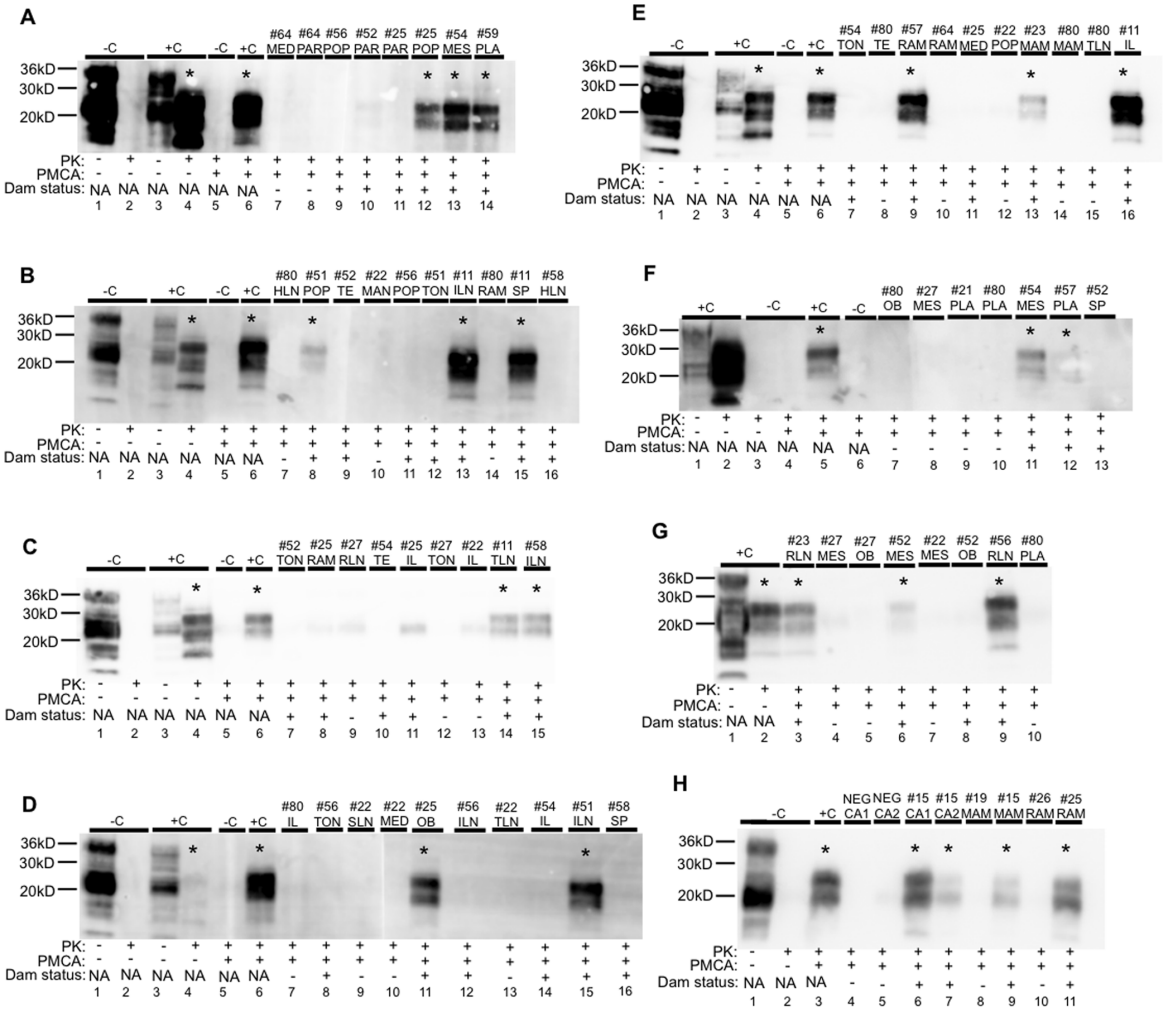
Amplifiable CWD prions detected in muntjac dam and fetal tissues. All panels show representative Western blot results after 5 rounds of sPMCA. Samples considered CWD positive are denoted with an asterisk (*). (A-H) Complete PK digestion of PrP^C^ is shown in negative deer brain controls (-C) without and with PMCA. PrP^CWD^ detection is shown in positive deer controls (+C) following PK digestion without and with PMCA. Tissue abbreviations are defined as follows: C = control, MED = mediastinal lymph node, PAR = parotid lymph node, POP = popliteal lymph node, MES = mesenteric lymph node, PLA = placenta, HLN = hepatic lymph node, TE = third eyelid, MAN = mandibular lymph node, TON = tonsil, ILN = ileocecocolic lymph node, RAM = recto-anal mucosa associated lymphoid tissue, SP = spleen, RLN = retropharyngeal lymph node, IL = ileum, TLN = tracheobronchial lymph node, SLN = sublumbar lymph node, OB = obex, MAM = mammary lymph node, CA = caruncle.

**Figure 5:**
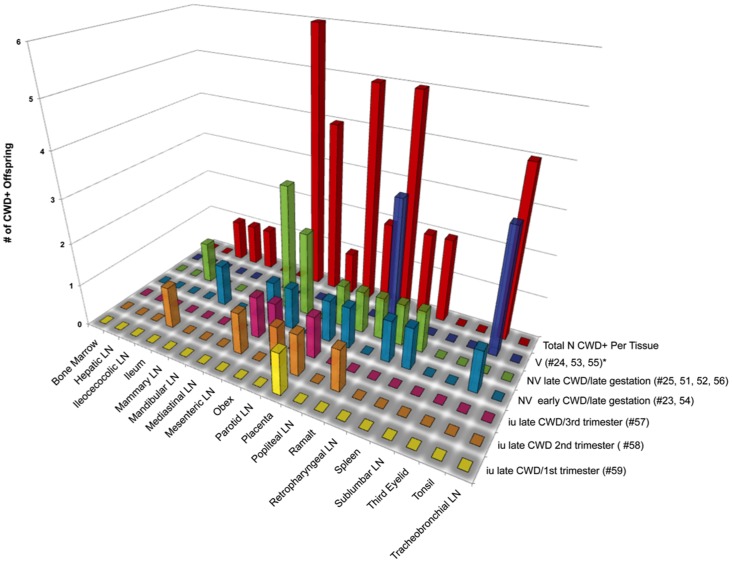
CWD detection in tissues harvested from offspring of CWD+ dams. Tonsilar and RAMALT biopsies harvested from viable (v) offspring were analyzed for PrP^CWD^ by IHC. All tissues from nonviable (NV) and *in utero* (IU) derived tissues were analyzed for amplifiable CWD prions by 5 rounds of sPMCA. Tissues from mock-inoculated offspring were IHC and or sPMCA negative.

PrP^CWD^ was first demonstrated by IHC in the lymphoid tonsil biopsy of offspring #24 at 40 dpb and the RAMALT biopsy at 102 dpb (Figure 3). Offspring #53 and #55 were IHC-PrP^CWD^ tonsil/RAMALT biopsy positive at 504/504 dpb and 465/386 dpb respectively (Figure 3). Amplifiable CWD prions were first detected in tonsil or RAMALT biopsy by 5 rounds of sPMCA in offspring #24 at 376 dpb, #53 at 107 dpb and #55 at 386 dpb (Figures 4 and 5). Lymphoid tonsil and RAMALT biopsies harvested from offspring of mock-inoculated controls (#20, #21, #22, #26) remained IHC and sPMCA PrP^CWD^ negative.

#### Full-term nonviable offspring

Amplifiable CWD prions were detected by sPMCA in 10 of 16 tissues tested from 6 full-term nonviable offspring (#23, #54, #25, #51, #52, #54, #56) born to CWD-infected dams (Table 2, Figures 4 and 5). All six nonviable offspring had at least two tissues containing amplifiable CWD prions– including obex, placenta, spleen, tonsil, RAMALT and popliteal, mammary, retropharyngeal, mesenteric and ileocecocolic lymph nodes. In two of the nonviable offspring (#52 and #56) that were born alive but died at 10 and 2 dpb, amplifiable CWD prions were demonstrated in retropharyngeal and mesenteric lymph node, spleen and RAMALT (Figures 4 and 5). Negative control tissues (#27) remained negative during 5 rounds of sPMCA.

**Table 2:** IHC and PMCA CWD detection in offspring born to mock and CWD-infected muntjac.

Offspring cohort	Dam’s CWD status	Dam #	Offspring #/status	Offspring IHC	Offspring PMCA
**Full term viable**	Positive	11	24/cl, fair 58 mpb	40*/102^∧^dpb	376^∧^dpb
		45	53/cl, term 45 mpb	504*^∧^dpb	107*/301^∧^dpb
		46	55/cl, term 40 mpb	465*/386^∧^dpb	386*^∧^dpb
	Negative	16	20	Negative	Negative
		16	22	ND	Negative
		19	21	ND	Negative
		19	26	Negative	Negative
**Full term nonviable**	Positive	11	23	ND	Pla, RLN, Sp, Ton, Pop, Mam
		50	54	ND	Ob, Mes
		15	25	ND	Ob, Pop
		47	51	ND	Ob, Pla, Mes, Iln, Pop
		44	52/died 10 dpb	ND	Mes, Sp, RAMALT
		43	56/died 2 dpb	ND	RLN, Mes
	Negative	16	27/died 5 dpb	ND	Negative
***In utero*** ** harvest**	Positive	47	57	ND	Ob, Pla, RAMALT
		45	58	ND	Pla, Mes, Iln, I, RAMALT, Par
		50	59	ND	Pla
	Negative	64	80	ND	Negative

**cl** = clinical; **fair** = health condition; * = tonsil biopsy; ^∧^ = RAMALT biopsy; **term** = terminated due to clinical TSE disease; **ND** = not done; **Pla** = placenta; **RLN** = retropharyngeal lymph node; **Sp** = spleen; **Ton** = tonsil; **Pop** = popliteal lymph node; **Mam** = mammary lymph node; **Ob** = obex; **Mes** = mesenteric lymph node; **Iln** = Ileocecocolic lymph node; **RAMALT** =  recto-anal mucosa associated lymphoid tissue; **I** = ileum; **Par** = parotid lymph node.

#### In utero harvested fetal tissues

Amplifiable CWD prions were detected by sPMCA in 6 of 10 tissues tested from 3 *in utero* harvested fetuses (#57, #58, #59) (Table 2, Figures 4 and 5). In all three (100%) fetuses at least one positive tissue– including obex, placenta, ileum, RAMALT, mesenteric and parotid lymph nodes (Figures 4 and 5) was demonstrated. *In utero* harvested negative control tissues (#80) remained negative during 5 rounds of sPMCA.

### Clinical Disease Progression in CWD-positive Muntjac Offspring

All three viable offspring born to CWD-infected dams developed clinical disease. Fawn #24 exhibited weight loss, muscle wasting and an unthrifty appearance at 28 mpb. Clinical disease progression in this CWD-infected fawn plateaued at 30 mpb and has not yet changed (currently at 50 mpb). Viable fawns #53 and #55 developed terminal disease including >20% weight loss, body mass loss, excessive salivation, rumen regurgitation and ataxia, and were euthanized at 34 and 31 mpb, respectively.

## Discussion

### Reeves’ Muntjac Deer are Susceptible to CWD Infection

CWD, the only prion disease in a wildlife population, exhibits high transmission rates among cervids in their natural environment. We and others (A. Young and R. Bessen, unpublished) demonstrated that PO and PO/SQ inoculated Reeves’ muntjac deer are susceptible to CWD infection, become lymphoid biopsy CWD-positive as early as 4 mpi and develop terminal disease in 18–24 months. Similar findings have been reported for other naturally and experimentally CWD-infected cervid species [38], [43], [56], [57]. In further support of the Reeves’ muntjac as a suitable host/model for CWD studies our CerTgPrP bioassay confirms that clinical disease presentation and time to terminal disease of muntjac CWD-inoculated mice is comparable to that of mule deer and elk-inoculated CerTgPrP mice (160–230 dpi) [55]. In this study we have demonstrated that a CWD susceptible deer species is able to maintain full term pregnancies throughout the course of disease. Our observations are congruent with those of Blanchong et al. [58] in white-tailed deer. We have shown that muntjac are a useful model for studying CWD.

### Transmission of CWD to Offspring

Maternally-associated transmission of prion diseases has been suggested for BSE [28]–[30], FSE [31], CJD [12] and scrapie [23], [59], [60]. For decades it has been known that scrapie incidence increases during the lambing season [14], [23]–[27]. As assay sensitivity improved, PrP^RES^ was detected in placentomes, placental and fetal tissues harvested from sheep naturally infected with scrapie [21], [22]. Also, the presence of CJD prions was demonstrated in brain, placenta and umbilical cord blood collected from an infected woman using mouse bioassays [12]. In this study, using both conventional assays and assays with heightened sensitivity, we have demonstrated for the first time CWD transmission from infected mothers to full term viable and nonviable offspring, as well as to *in utero* fetuses.

#### Viable offspring

The generation of a cohort of offspring that carry and potentially shed infectious prions to the environment and/or one another is of particular interest due to the efficiency of CWD transmission in the native host population. In this study we have demonstrated mother to offspring transmission and disease progression to a viable cohort of offspring that were born to CWD-infected dams. This is the earliest demonstration of TSE IHC tonsil biopsy positivity. Of interest is that all three fawns surpassed the time when terminal disease is typically observed (18–24 months) in cervid species [43], [61], [62]. It is possible that these fawns were exposed to a very low dose of maternal infectious prions or that a unique strain is associated with mother to offspring transmission. The single remaining CWD positive viable offspring will continue to be monitored for clinical disease progression, development of blood prionemia, as well as ability to procreate and transmit disease to its own offspring.

#### Nonviable offspring

In our study, we were able to closely monitor breeding, pregnancy and delivery. We observed a 60% decrease in full-term viable offspring born to CWD-infected dams when compared to naïve controls. We have demonstrated that 100% of these nonviable offspring have at least two tissues containing amplifiable CWD prions. In addition, we have demonstrated amplifiable CWD prions in tissues harvested from offspring that were born alive but failed to thrive and died a few days post birth, suggesting *in utero* exposure.

### Transmission of CWD *in utero*

Assessing prion infection *in utero* has been challenging due to limitations in assay sensitivity. Recently, Garza et al. [22] demonstrated amplifiable prions in fetal and maternal tissues harvested from sheep naturally infected with scrapie. Here, using the same highly sensitive PMCA assay, we have analyzed early and late gestational stage fetal tissues harvested from CWD-infected dams in the early (< ½ the time to terminal CWD) or late (> ½ the time to terminal disease) stage of CWD infection and found that 100% of *in utero* harvested fetuses contain amplifiable CWD prions. Detection of PrP^CWD^ in early gestational fetal tissues indicates the existence of a mechanism for vertical prion transmission.

### Potential Mechanisms for Maternal to Fetal Transmission

Infected ewes harbor scrapie prions in mammary tissues [63] and can transmit infection to lambs that ingest their milk [64]. Tuo et al. [21], Andreoletti et al. [17] and Tamai et al. [12] have provided additional evidence for vertical transmission by demonstrating prions in maternal and fetal tissues of sheep and humans. Of interest is the path infectious prions take from mother to offspring. Since a number of studies proved that infectious prions are present in the blood of symptomatic and asymptomatic hosts [4], [46]–[48], [65]–[67], it is possible that infectious prions are trafficked via maternal blood to fetal trophectoderm, where they are taken up by fetal-derived multinucleated trophoblast cells – cells with mobility and phagocytic properties [68]–[72] at the feto-maternal interface.

In addition, during gestation, to further the development of the fetal respiratory and digestive systems, the fetus recirculates approximately 500 ml of amniotic fluid daily via oronasal ingestion [73]–[75]. Amniotic fluid consists of maternal plasma and/or fetal urine, depending upon gestational stage (primarily maternal plasma in early gestation vs. mostly fetal urine in late gestation) [76]. If either the amniotic fluid or the fetus contains infectious prions, susceptible fetal mucosal surfaces could be continuously bathed in – and thus exposed to – infectious prions throughout gestation.

### Implications Associated with Transmission of CWD to Offspring

The facile transmission of CWD seen in herds of free ranging and captive cervids has led to questions regarding the modes of transmission. While direct contact with infected cervids, as well as indirect contact with infectious bodily fluids and contaminated environments are suspected to account for much of this transmission, a third path of transmission, the one from mother to offspring, may be underappreciated. PrP^CWD^ placental accumulation and CWD *in utero* acquirement would help explain the surprisingly high rates of CWD among cervids. Moreover, CWD positive nonviable offspring carcasses 1) would contribute to environmental prion contamination [77] and 2) would be available for consumption by scavenger species, thus increasing the potential for cross-species transmission [53], [78], [79]. Viable offspring may spread disease by 1) direct cervid-to-cervid contact, 2) multigenerational mother to offspring transmission, as well as 3) shedding infectious prions in bodily secretions (urine, feces, saliva, blood) during their entire lifespan.

Here, in an experimental model of CWD, we have demonstrated the transmission of infectious prions from clinical and subclinical mothers to full-term viable, nonviable and *in utero* harvested offspring, revealing that the transmission of TSEs from mother to offspring can occur and may be underestimated for all prion diseases.
